# Association between red blood cell distribution width to albumin ratio (RAR) and cataract risk: A cross-sectional study from NHANES 1999–2008

**DOI:** 10.1016/j.aopr.2025.11.007

**Published:** 2025-12-01

**Authors:** Renfang Zhang, Fuyao Chen, Yuxin Huang, Xingchao Shentu

**Affiliations:** a Eye Center, The Second Affiliated Hospital, Zhejiang University School of Medicine, Hangzhou, China; b Zhejiang Provincial Key Laboratory of Ophthalmology, Zhejiang Provincial Clinical Research Center for Eye Diseases, Zhejiang Provincial Engineering Institute on Eye Diseases, Hangzhou, China

**Keywords:** Cataract, RAR, Systemic inflammation, Nutritional deficiency, Epidemiology

## Abstract

**Objective:**

To evaluate the association between red blood cell distribution width to albumin ratio (RAR) and cataract risk among US adults.

**Methods:**

A cross-sectional study was conducted utilizing the National Health and Nutrition Examination Survey (NHANES) from 1999 to 2008. RAR was calculated using red blood cell distribution width and serum albumin measurements obtained from mobile examination centers, with higher values indicating greater systemic inflammation and nutritional deficiency. Self-reported history of cataract surgery served as a proxy for cataract diagnosis. Covariates included sociodemographic factors, lifestyle variables, and comorbidities. Multivariable logistic regression models were employed to evaluate the association between RAR and cataract. Restricted cubic spline (RCS) analysis was performed to examine potential nonlinear relationships. Propensity score matching (PSM) was implemented to reduce confounding bias. Finally, subgroup analysis was performed to investigate potential interaction effects.

**Results:**

The analytical cohort comprised 26397 participants, among whom 2295 (8.70%) had cataract. Cataract patients exhibited significantly higher RAR values (3.22 ± 0.47) compared to non-cataract participants (3.06 ± 0.46). After adjustment for all covariates, a positive association between RAR and cataract was observed, both when RAR was treated as a continuous variable (odds ratio (OR)=1.412, 95% confidence interval (CI): 1.208–1.649; *P* <0.001) and when analyzed categorically by quartiles. Participants in the highest RAR quartile had significantly increased cataract risk relative to the lowest quartile (OR=1.572, 95% CI: 1.261–1.958; *P* <0.001). RCS analysis revealed no evidence of nonlinearity (*P* for nonlinearity =0.520). This positive association persisted in a 1:2 PSM analysis. Subgroup analysis revealed no significant interaction effects across covariates, with the RAR–cataract association remaining statistically significant in most subgroups.

**Conclusions:**

Elevated RAR is independently associated with higher cataract prevalence, implicating systemic inflammation and nutritional deficiency as modifiable risk factors.

## 1 Introduction

Cataract represents the leading global cause of vision loss, characterized by lens opacification resulting from protein denaturation, which clinically manifests as progressive visual blurring, glare sensitivity, and diminished color discrimination.^1^ Although surgical intervention is a well-established treatment that can typically prevent cataract-related blindness,^2^ substantial barriers persist in low- and middle-income countries where patients often face delayed or inaccessible surgery due to financial constraints and limited healthcare resources.^3^, ^4^, ^5^, ^6^ Consequently, preventing cataract development is crucial for mitigating its significant health and socioeconomic burden.

Beyond the non-modifiable risk factor of aging, modifiable risk factors such as diabetes mellitus, ultraviolet radiation exposure, and smoking constitute key targets for cataract prevention and control.^7^, ^8^, ^9^, ^10^ Emerging evidence implicates systemic chronic inflammation in cataract pathogenesis, where inflammatory states activate oxidative stress pathways, thereby accelerating lens protein denaturation and aggregation.^11^^,^^12^ Red blood cell distribution width to albumin ratio (RAR) is an emerging composite biomarker calculated as the ratio of red blood cell distribution width (RDW) to serum albumin concentration. RAR levels increase under conditions of chronic inflammation, oxidative stress, and nutritional deficiency.^13^^,^^14^ Robust literature demonstrates the superior prognostic and risk stratification value of RAR across multiple chronic diseases including age-related macular degeneration, diabetic retinopathy, cardiovascular events, cancer and depression.^15^, ^16^, ^17^, ^18^, ^19^, ^20^

Notably, the association between RAR and cataract development remains unexplored. This study utilized cross-sectional data from the 1999–2008 National Health and Nutrition Examination Survey (NHANES) to evaluate the relationship between RAR and cataract prevalence among US adults. Our findings may provide a novel approach for early screening and intervention in populations at elevated cataract risk.

## 2 Materials and methods

### 2.1 Data source and subject selection

The NHANES is a national survey from the United States designed to collect comprehensive health and nutritional status data from people of all ages in the US, conducted by the National Center for Health Statistics (NCHS).^21^ It employs a complex, stratified, multistage probability cluster sampling method to ensure accurate representation of the diverse population. Detailed information about this database is available at http://www.cdc.gov/nchs/nhanes/. Ethical approval for the study was granted by the Ethics Committee of the National Center for Health Statistics (Protocol #98-12 for 1999–2004, Protocol #2005-06 for 2005–2008). All subjects signed written informed consent. In this study, we used data from five consecutive cycles (1999–2008) of the NHANES database. Participants were excluded if they met any of the following criteria: (1) aged less than 20 years; (2) missing cataract information; (3) missing RAR data; (4) missing covariate data. Finally, a total of 26397 survey participants were included in our analysis.

### 2.2 Cataract identification criteria

According to the NHANES Vision Procedures Manual, participants aged over 20 years were asked: "Have you ever had cataract surgery?" An affirmative response was considered indicative of having cataract. This diagnostic approach aligns with methods employed in prior studies,^22^, ^23^, ^24^ and is consistent with the Preferred Practice Pattern® Guidelines for Cataract in the Adult Eye published by the American Academy of Ophthalmology,^25^ given the high accessibility and low barriers to cataract surgery in the United States.

### 2.3 Calculation of RAR

RDW (%) was measured in NHANES mobile examination centers using a Coulter analyzer (Beckman Coulter, Brea, CA) through peripheral blood samples, employing the manufacturer's proprietary counting and sizing methodology.^21^ Serum albumin concentration was quantified using the LX20 system (Beckman Coulter) via the bichromatic digital endpoint method. In this assay, albumin binds with bromocresol purple (BCP) reagent to form a colored complex. The system monitors absorbance change at 600 nm, with the magnitude of change being directly proportional to albumin concentration in the sample. RAR was calculated using the formula^14^: RAR=RDW(%)/Albumin(g/dL)

### 2.4 Covariates assessment

Covariates were selected based on established cataract epidemiology literature,^26^, ^27^, ^28^ with sociodemographic variables collected via computer-assisted personal interviews including age (years), gender (male/female), race/ethnicity (Non-Hispanic White/Non-Hispanic Black/Mexican American/Other), education level (less than high school/high school or above), marital status (married or living with partner/unmarried or other), economic status (family income-to-poverty ratio <1.00/≥1.00), and body mass index (BMI, calculated as weight in kg/height in m^2^ and further categorized into <18.5/18.5–25/≥25 kg/m^2^). Comorbidities assessed included hypertension (defined as a physician's diagnosis, use of antihypertensive medication, or systolic blood pressure ≥140 mmHg and/or diastolic blood pressure ≥90 mmHg), hyperlipidemia (defined as a physician's diagnosis, use of cholesterol-lowering medication, or total cholesterol ≥240 mg/dL), and diabetes mellitus (defined as a physician's diagnosis, use of glucose-lowering medication/insulin, or HbA_1_c ≥6.5%). Lifestyle factors comprised smoking categorized as non-smokers (<100 lifetime cigarettes), former smokers (≥100 cigarettes but had quit smoking by the time of interview), or current smokers (≥100 cigarettes and currently smoking), and alcohol consumption classified as non-drinkers (<12 lifetime drinks) and drinkers (≥12 drinks).

### 2.5 Statistical analysis

NHANES employed a complex stratified multistage sampling design to ensure national representativeness of the US population. Our analysis incorporated sampling weights, specifically selecting subsample weights corresponding to the minimal subsample according to Centers for Disease Control and Prevention (CDC) guidelines.^29^ All required data were extracted from publicly available NHANES data files for each cycle and then merged using the unique participant identifier to form a single analytic dataset. Continuous variables were presented as mean ± standard deviation (SD), while categorical variables are expressed as frequencies with percentages (%). RAR was analyzed both as a continuous variable and categorically by quartiles (Q). Between-group comparisons utilized Student's *t*-tests for continuous variables and *χ*^2^ tests for categorical variables. Three weighted logistic regression models were constructed to examine the association between RAR and cataract: Model 1 (unadjusted), Model 2 (adjusted for age, gender, and race/ethnicity), and Model 3 (further adjusted for all covariates). Results are reported as odds ratios (ORs) with 95% confidence intervals (CIs). All variance inflation factors (VIFs) in the three models were less than 2, confirming no significant multicollinearity. To investigate potential nonlinear relationships, restricted cubic spline (RCS) models with 3 knots were implemented, with full covariate adjustment in RCS analyses. The number of knots was determined by Akaike Information Criterion (AIC) minimization, and the final model used three knots placed at the 10th, 50th, and 90th percentiles of RAR distribution. Given significant covariate differences across RAR quartiles, a 1:2 propensity score matching (PSM) analysis including all covariates was performed to mitigate confounding bias. The propensity scores were estimated using a generalized linear model, and nearest-neighbor matching was applied to pair participants between two groups. Subgroup analysis stratified by all covariates was conducted to examine potential interaction effects. All statistical analyses and visualizations were performed using R software (version 4.5.0, R Foundation for Statistical Computing, Austria). A two-tailed *P* value <0.05 was considered statistically significant.

## 3 Results

### 3.1 Study population characteristics

**Fig. 1:**
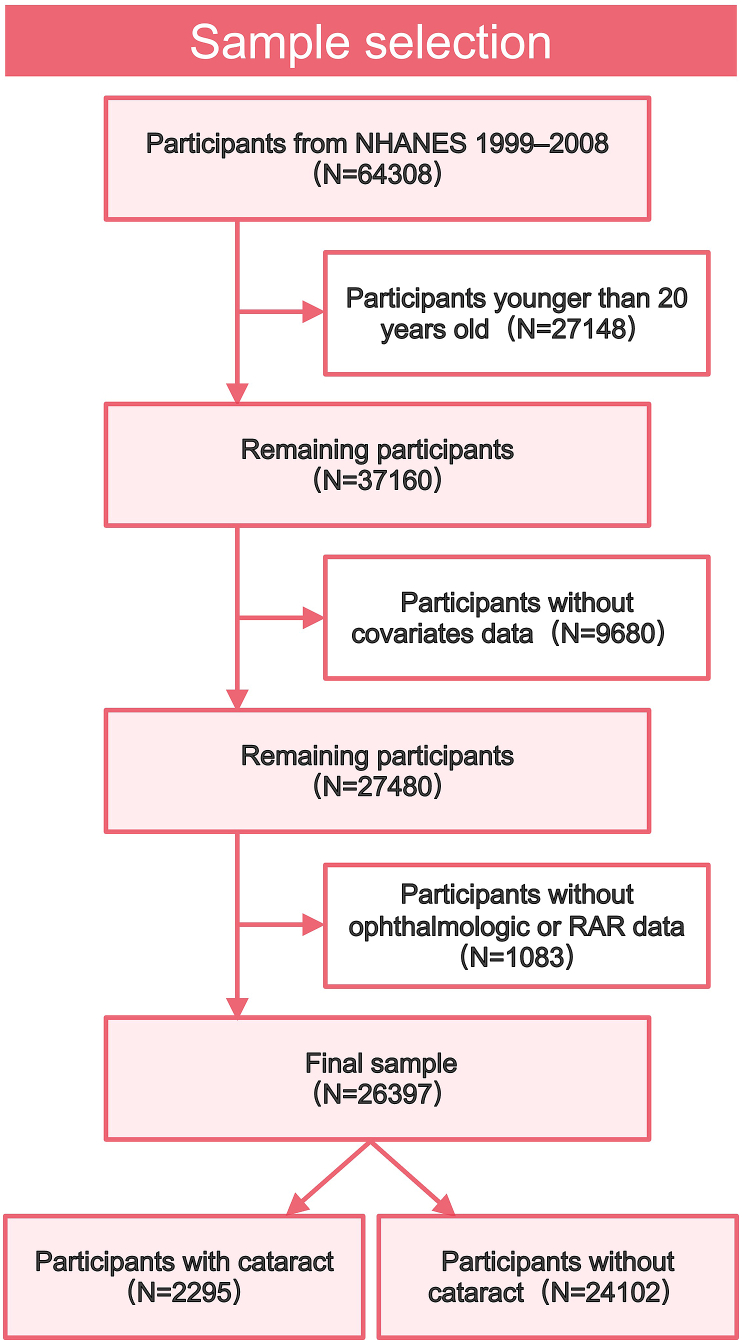
Flowchart of the study population. Participants younger than 20 years old or without necessary information for analysis were excluded from the study. Finally, 26397 participants were enrolled and further classified as cataract or non-cataract group.

The sample selection process was shown in Fig. 1. The study population comprised 26397 participants with a mean age of 49.5 years, including 13009 males (49.3%) and 13388 females (50.7%). Cataract was present in 2295 participants (8.70%). Generally, all variables except BMI demonstrated significant differences between groups (*P* <0.001). As presented in Table 1, females, non-Hispanic White individuals, those with lower educational attainment, unmarried participants, and individuals with relatively better economic status demonstrated a higher likelihood of developing cataract. Participants with a history of smoking, alcohol consumption, or comorbidities also exhibited elevated cataract risk. Consistent with our initial hypothesis, cataract patients showed significantly higher RAR levels. To further examine the relationship between RAR and covariates, we constructed an additional baseline characteristics table categorizing participants by RAR quartiles. As shown in Table 2, the proportions of individuals with obesity, hypertension, diabetes, hyperlipidemia, and cataract significantly increased across ascending RAR quartiles. These overall trends and proportions aligned with the conclusions derived from Table 1.

**Table 1:** Characteristics of participants stratified by cataract (NHANES 1999–2008).

	All	**Non-cataract**	Cataract	*P* value
	N=26397	N=24102	N=2295	
**Gender (N, %)**				<0.001
Male	13009 (49.3%)	11960 (49.6%)	1049 (45.7%)	
Female	13388 (50.7%)	12142 (50.4%)	1246 (54.3%)	
**Age (years, mean (SD))**	49.5 (18.3)	47.1 (17.2)	74.8 (9.02)	<0.001
**Race (N, %)**				<0.001
Mexican American	5262 (19.9%)	5037 (20.9%)	225 (9.80%)	
Non-Hispanic White	13635 (51.7%)	12015 (49.9%)	1620 (70.6%)	
Non-Hispanic Black	4911 (18.6%)	4631 (19.2%)	280 (12.2%)	
Other	2589 (9.81%)	2419 (10.0%)	170 (7.41%)	
**Education (N, %)**				<0.001
Less than high school	7677 (29.1%)	6782 (28.1%)	895 (39.0%)	
High school or above	18720 (70.9%)	17320 (71.9%)	1400 (61.0%)	
**Marital status (N, %)**				<0.001
Married or living with partner	16608 (62.9%)	15387 (63.8%)	1221 (53.2%)	
Unmarried or other	9789 (37.1%)	8715 (36.2%)	1074 (46.8%)	
**Economic status (N, %)**				<0.001
Below poverty	4411 (16.7%)	4092 (17.0%)	319 (13.9%)	
Above poverty	21986 (83.3%)	20010 (83.0%)	1976 (86.1%)	
**Body mass index (N, %)**				0.354
<18.5	402 (1.52%)	368 (1.53%)	34 (1.48%)	
18.5–25	7559 (28.6%)	6872 (28.5%)	687 (29.9%)	
≥25	18436 (69.8%)	16862 (70.0%)	1574 (68.6%)	
**Smoking (N, %)**				<0.001
Non-smoker	13503 (51.2%)	12429 (51.6%)	1074 (46.8%)	
Current smoker	5861 (22.2%)	5634 (23.4%)	227 (9.89%)	
Former smoker	7033 (26.6%)	6039 (25.1%)	994 (43.3%)	
**Alcohol consumption (N, %)**				<0.001
Non-drinker	18492 (70.1%)	17150 (71.2%)	1342 (58.5%)	
Drinker	7905 (29.9%)	6952 (28.8%)	953 (41.5%)	
**Hypertension (N, %)**				<0.001
Yes	10614 (40.2%)	8991 (37.3%)	1623 (70.7%)	
No	15783 (59.8%)	15111 (62.7%)	672 (29.3%)	
**Hyperlipidemia (N, %)**				<0.001
Yes	8872 (33.6%)	7810 (32.4%)	1062 (46.3%)	
No	17525 (66.4%)	16292 (67.6%)	1233 (53.7%)	
**Diabetes mellitus (N, %)**				<0.001
Yes	3830 (14.5%)	3141 (13.0%)	689 (30.0%)	
No	22567 (85.5%)	20961 (87.0%)	1606 (70.0%)	
**RAR (mean (SD))**	3.07 (0.47)	3.06 (0.47)	3.22 (0.46)	<0.001
**RAR quartile (N, %)**				<0.001
Q1	6621 (25.1%)	6373 (26.4%)	248 (10.8%)	
Q2	6598 (25.0%)	6127 (25.4%)	471 (20.5%)	
Q3	6586 (24.9%)	5907 (24.5%)	679 (29.6%)	
Q4	6592 (25.0%)	5695 (23.6%)	897 (39.1%)

**Table 2:** Characteristics of participants stratified by RAR (NHANES 1999–2008).

	All	Q1	Q2	Q3	Q4	*P* value
	N=26397	N=6621	N=6598	N=6586	N=6592	
**Gender (N, %)**						<0.001
Male	13009 (49.3%)	4322 (65.3%)	3648 (55.3%)	2923 (44.4%)	2116 (32.1%)	
Female	13388 (50.7%)	2299 (34.7%)	2950 (44.7%)	3663 (55.6%)	4476 (67.9%)	
**Age (years, mean (SD))**	49.5 (18.3)	42.5 (16.5)	49.5 (17.4)	53.3 (18.0)	52.7 (19.4)	<0.001
**Race (N, %)**						<0.001
Mexican American	5262 (19.9%)	1510 (22.8%)	1381 (20.9%)	1266 (19.2%)	1105 (16.8%)	
Non-Hispanic White	13635 (51.7%)	3818 (57.7%)	3590 (54.4%)	3422 (52.0%)	2805 (42.6%)	
Non-Hispanic Black	4911 (18.6%)	606 (9.15%)	942 (14.3%)	1273 (19.3%)	2090 (31.7%)	
Other	2589 (9.81%)	687 (10.4%)	685 (10.4%)	625 (9.49%)	592 (8.98%)	
**Education (N, %)**						<0.001
Less than high school	7677 (29.1%)	1606 (24.3%)	1868 (28.3%)	2004 (30.4%)	2199 (33.4%)	
High school or above	18720 (70.9%)	5015 (75.7%)	4730 (71.7%)	4582 (69.6%)	4393 (66.6%)	
**Marital status (N, %)**						<0.001
Married or living with partner	16608 (62.9%)	4394 (66.4%)	4376 (66.3%)	4088 (62.1%)	3750 (56.9%)	
Unmarried or other	9789 (37.1%)	2227 (33.6%)	2222 (33.7%)	2498 (37.9%)	2842 (43.1%)	
**Economic status (N, %)**						<0.001
Below poverty	4411 (16.7%)	903 (13.6%)	1066 (16.2%)	1100 (16.7%)	1342 (20.4%)	
Above poverty	21986 (83.3%)	5718 (86.4%)	5532 (83.8%)	5486 (83.3%)	5250 (79.6%)	
**Body mass index (N, %)**						<0.001
<18.5	402 (1.52%)	151 (2.28%)	100 (1.52%)	84 (1.28%)	67 (1.02%)	
18.5–25	7559 (28.6%)	2595 (39.2%)	1927 (29.2%)	1686 (25.6%)	1351 (20.5%)	
≥25	18436 (69.8%)	3875 (58.5%)	4571 (69.3%)	4816 (73.1%)	5174 (78.5%)	
**Smoking (N, %)**						0.001
Non-smoker	13503 (51.2%)	3400 (51.4%)	3327 (50.4%)	3350 (50.9%)	3426 (52.0%)	
Current smoker	5861 (22.2%)	1549 (23.4%)	1465 (22.2%)	1489 (22.6%)	1358 (20.6%)	
Former smoker	7033 (26.6%)	1672 (25.3%)	1806 (27.4%)	1747 (26.5%)	1808 (27.4%)	
**Alcohol consumption (N, %)**						<0.001
Non-drinker	18492 (70.1%)	5254 (79.4%)	4821 (73.1%)	4464 (67.8%)	3953 (60.0%)	
Drinker	7905 (29.9%)	1367 (20.6%)	1777 (26.9%)	2122 (32.2%)	2639 (40.0%)	
**Hypertension (N, %)**						<0.001
Yes	10614 (40.2%)	2049 (30.9%)	2497 (37.8%)	2978 (45.2%)	3090 (46.9%)	
No	15783 (59.8%)	4572 (69.1%)	4101 (62.2%)	3608 (54.8%)	3502 (53.1%)	
**Hyperlipidemia (N, %)**						<0.001
Yes	8872 (33.6%)	1923 (29.0%)	2198 (33.3%)	2338 (35.5%)	2413 (36.6%)	
No	17525 (66.4%)	4698 (71.0%)	4400 (66.7%)	4248 (64.5%)	4179 (63.4%)	
**Diabetes mellitus (N, %)**						<0.001
Yes	3830 (14.5%)	542 (8.19%)	791 (12.0%)	1065 (16.2%)	1432 (21.7%)	
No	22567 (85.5%)	6079 (91.8%)	5807 (88.0%)	5521 (83.8%)	5160 (78.3%)	
**Cataract (N, %)**						<0.001
Non-cataract	24102 (91.3%)	6373 (96.3%)	6127 (92.9%)	5907 (89.7%)	5695 (86.4%)	
Cataract	2295 (8.69%)	248 (3.75%)	471 (7.14%)	679 (10.3%)	897 (13.6%)	

### 3.2 Association between RAR and cataract

**Fig. 2:**
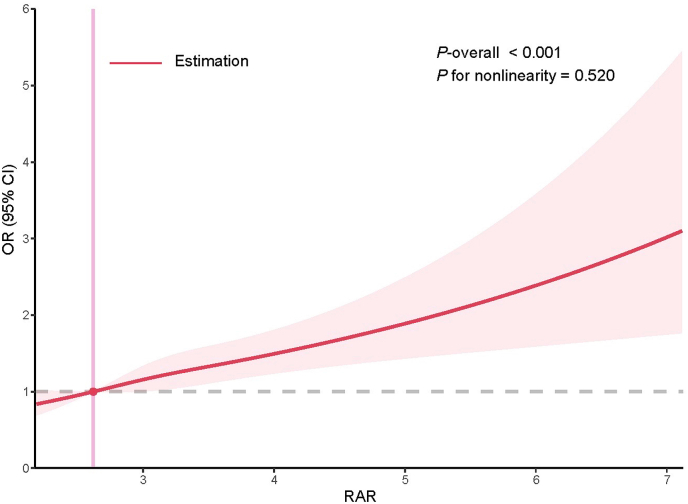
RCS analysis of the association between RAR and cataract. Three knots were placed at the 10th, 50th, and 90th percentiles of RAR. The model was adjusted for all covariates, including age, gender, race/ethnicity, education level, marital status, economic status, BMI, smoking, alcohol consumption, hypertension, hyperlipidemia and diabetes mellitus. The red line and the pink area stand for the estimated OR and 95% confidence intervals.

A subsequent weighted multivariable logistic regression analysis demonstrated a significant positive association between elevated RAR and cataract prevalence (Table 3). This association remained significant across all analytical models: Model 1 (unadjusted: OR=2.390, 95% CI: 2.128–2.685; *P* <0.001), Model 2 (partially adjusted: OR=1.545, 95% CI: 1.328–1.797; *P* <0.001), and Model 3 (fully adjusted: OR=1.412, 95% CI: 1.208–1.649; *P* <0.001). We next analyzed RAR as a categorical variable by quartiles, which confirmed this positive relationship. Notably, even after progressive covariate adjustment, participants in the highest RAR quartile (Q4) had significantly higher odds of cataract compared to the lowest quartile (Q1) in both Model 2 (OR=1.711, 95% CI: 1.381–2.119; *P* <0.001) and Model 3 (OR=1.572, 95% CI: 1.261–1.958; *P* <0.001). To investigate potential nonlinearity, restricted cubic spline (RCS) analysis was further performed. As visualized in Fig. 2, a monotonically increasing curve indicated a dose-response relationship between RAR and cataract risk, and no significant nonlinear association was observed (*P* for nonlinearity =0.520).

**Table 3:** Association between RAR and cataract by logistic regression.

	Model 1	Model 2	Model 3
RAR	2.390 (2.128–2.685)	1.545 (1.328–1.797)	1.412 (1.208–1.649)
	<0.001	<0.001	<0.001
RAR quartile			
Q1	reference	reference	reference

Q2	1.877 (1.545–2.280)	0.930(0.729–1.186)	0.952 (0.745–1.217)
	<0.001	0.551	0.689
Q3	3.015 (2.557–3.555)	1.176 (0.935–1.480)	1.139 (0.874–1.485)
	<0.001	0.163	0.329
Q4	4.972(4.176–5.919)	1.711 (1.381–2.119)	1.572 (1.261–1.958)
	<0.001	<0.001	<0.001

Note: Model 1: no covariates were adjusted. Model 2: adjusted for age, gender and race/ethnicity. Model 3: adjusted for age, gender, race/ethnicity, education level, marital status, economic status, BMI, smoking, alcohol consumption, hypertension, hyperlipidemia and diabetes mellitus.

### 3.3 PSM analysis

Given significant covariate variations across RAR strata, a propensity score matching (PSM) analysis was conducted at a 1:2 ratio. After matching, the study population consisted of 6330 participants, with 2110 in the cataract group and 4220 in the non-cataract group. Table 4 presented baseline characteristics of the matched population, revealing significantly higher RAR levels in the cataract group compared to non-cataract controls (*P* <0.001). Subsequent logistic regression analysis confirmed a consistent positive association between elevated RAR and cataract incidence across all three models: Model 1 (OR=1.624, 95% CI: 1.332–1.980), Model 2 (OR=1.481, 95% CI: 1.222–1.795), and Model 3 (OR=1.429, 95% CI: 1.189–1.717). When analyzed categorically, participants in the highest quartile (Q4) demonstrated significantly increased cataract incidence compared to the lowest quartile (Q1) reference group (*P* <0.001), as detailed in Table 5.

**Table 4:** Characteristics of participants after 1:2 PSM

	All	**Non-cataract**	Cataract	*P* value
	N=6330	N=4220	N=2110	
**Gender (N, %)**				0.003
Male	3098 (48.9%)	2122 (50.3%)	976 (46.3%)	
Female	3232 (51.1%)	2098 (49.7%)	1134 (53.7%)	
**Age (years, mean (SD))**	72.4 (8.62)	71.3 (8.15)	74.6 (9.10)	<0.001
**Race (N, %)**				<0.001
Mexican American	737 (11.6%)	528 (12.5%)	209 (9.91%)	
Non-Hispanic White	4240 (67.0%)	2736 (64.8%)	1504 (71.3%)	
Non-Hispanic Black	882 (13.9%)	626 (14.8%)	256 (12.1%)	
Other	471 (7.44%)	330 (7.82%)	141 (6.68%)	
**Education (N, %)**				0.310
Less than high school	2409 (38.1%)	1587 (37.6%)	822 (39.0%)	
High school or above	3921 (61.9%)	2633 (62.4%)	1288 (61.0%)	
**Marital status (N, %)**				<0.001
Married or living with partner	3686 (58.2%)	2546 (60.3%)	1140 (54.0%)	
Unmarried or other	2644 (41.8%)	1674 (39.7%)	970 (46.0%)	
**Economic status (N, %)**				0.348
Below poverty	997 (15.8%)	678 (16.1%)	319 (15.1%)	
Above poverty	5333 (84.2%)	3542 (83.9%)	1791 (84.9%)	
**Body mass index (N, %)**				0.019
<18.5	91 (1.44%)	60 (1.42%)	31 (1.47%)	
18.5–25	1732 (27.4%)	1108 (26.3%)	624 (29.6%)	
≥25	4507 (71.2%)	3052 (72.3%)	1455 (69.0%)	
**Smoking (N, %)**				0.033
Non-smoker	2882 (45.5%)	1904 (45.1%)	978 (46.4%)	
Current smoker	717 (11.3%)	509 (12.1%)	208 (9.86%)	
Former smoker	2731 (43.1%)	1807 (42.8%)	924 (43.8%)	
**Alcohol consumption (N, %)**				0.012
Non-drinker	3863 (61.0%)	2622 (62.1%)	1241 (58.8%)	
Drinker	2467 (39.0%)	1598 (37.9%)	869 (41.2%)	
**Hypertension (N, %)**				0.237
Yes	4377 (69.1%)	2897 (68.6%)	1480 (70.1%)	
No	1953 (30.9%)	1323 (31.4%)	630 (29.9%)	
**Hyperlipidemia (N, %)**				0.142
Yes	2979 (47.1%)	2014 (47.7%)	965 (45.7%)	
No	3351 (52.9%)	2206 (52.3%)	1145 (54.3%)	
**Diabetes mellitus (N, %)**				0.127
Yes	1799 (28.4%)	1173 (27.8%)	626 (29.7%)	
No	4531 (71.6%)	3047 (72.2%)	1484 (70.3%)	
**RAR (mean (SD))**	3.16 (0.45)	3.14 (0.44)	3.21 (0.46)	<0.001
**RAR quartile (N, %)**				<0.001
Q1	852 (13.5%)	623 (14.8%)	229 (10.9%)	
Q2	1432 (22.6%)	990 (23.5%)	442 (20.9%)	
Q3	1943 (30.7%)	1318 (31.2%)	625 (29.6%)	
Q4	2103 (33.2%)	1289 (30.5%)	814 (38.6%)

**Table 5:** Association between RAR and cataract in PSM analysis.

	Model 1	Model 2	Model 3
RAR	1.624 (1.332–1.980)	1.481 (1.222–1.795)	1.429 (1.189–1.717)
	<0.001	<0.001	<0.001
RAR quartile			
Q1	reference	reference	reference

Q2	1.169 (0.866–1.579)	1.048 (0.771–1.424)	1.032 (0.754–1.414)
	0.302	0.760	0.836
Q3	1.358 (1.019–1.810)	1.150 (0.861–1.537)	1.137 (0.846–1.528)
	0.037	0.338	0.387
Q4	1.977 (1.562–2.503)	1.650 (1.284–2.121)	1.594 (1.235–2.056)
	<0.001	<0.001	<0.001

Note: Model 1: no covariates were adjusted. Model 2: adjusted for age, gender and race/ethnicity. Model 3: adjusted for age, gender, race/ethnicity, education level, marital status, economic status, BMI, smoking, alcohol consumption, hypertension, hyperlipidemia and diabetes mellitus.

### 3.4 Subgroup analysis

**Fig. 3:**
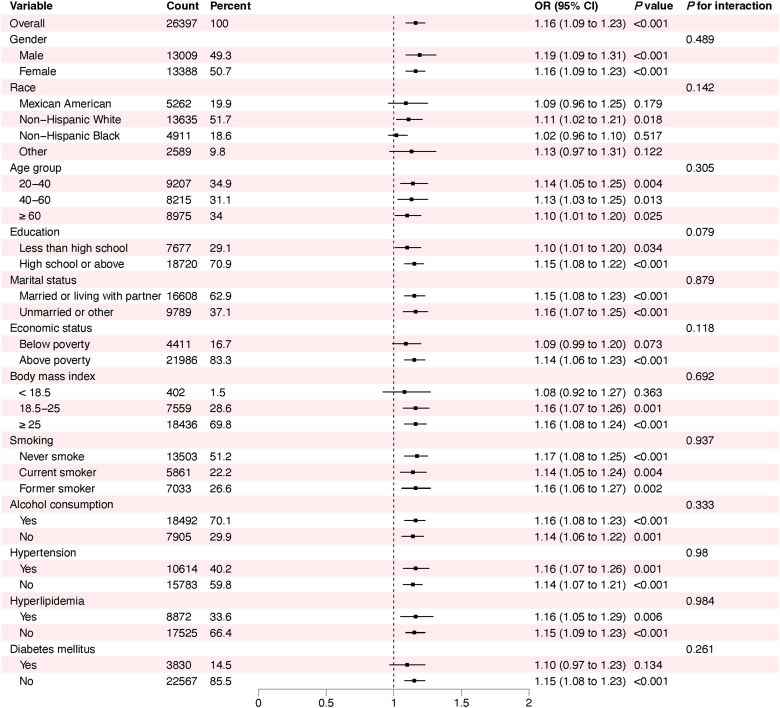
Forest plot for the results in subgroup analysis. The black dot and the horizontal black line represent the OR and its 95% confidence interval, respectively.

To assess potential effect modification and determine whether the RAR-cataract association varied across population characteristics, we performed subgroup analysis using the fully adjusted logistic regression model (Model 3) for all covariates. As summarized in Fig. 3, no significant interaction effects were observed between RAR and any covariate (*P* >0.05). Crucially, a significant positive association between elevated RAR and cataract incidence persisted in the majority of subgroups (*P* <0.05).

## 4 Discussion

This study established a significant positive association between elevated RAR and cataract risk using the NHANES database, constituting the first investigation exploring this relationship and providing novel evidence supporting the impact of systemic inflammation and nutritional status on cataractogenesis.

To interpret these findings in a biological context, we reviewed existing evidence on inflammation and cataractogenesis. The link between cataract and inflammation has been well-documented. Local inflammatory mediators can induce oxidative stress, which promotes crystallin protein cross-linking and denaturation. For example, inadequately treated uveitis carries a cataract incidence probability as high as 18%–49%, representing its most common vision-threatening complication.^30^ In pediatric uveitis patients, aqueous humor inflammatory cytokine levels exhibit a dose-dependent relationship with complicated cataract occurrence^31^; in diabetic patients, the hyperglycemic microenvironment activates the TXNIP/NLRP3 inflammasome pathway in lens epithelial cells, triggering apoptosis and subsequent cataract formation.^32^ Notably, the role of systemic chronic inflammation in cataract pathogenesis is increasingly recognized. Multiple prospective cohort studies and retrospective analyses indicate that pro-inflammatory dietary patterns (characterized by high red meat and refined grain intake) and elevated Dietary Inflammatory Index increase the risk of age-related cataract development.^33^, ^34^, ^35^ Other cross-sectional studies demonstrate that elevated systemic immune-inflammation index (SII) and systemic inflammatory response index (SIRI) correlate with increased cataract incidence.^36^^,^^37^ Furthermore, compared to healthy individuals, cataract surgery patients exhibit higher urinary levels of 8-iso-prostaglandin F_2_α, which is a biomarker measuring systemic oxidative stress and inflammation levels,^38^ while showing no significant differences in serum IL-6 and CRP concentrations.^39^^,^^40^ These discordant biomarker findings illustrate the limitations of single biological markers in reflecting systemic inflammatory status. Consequently, identifying a robust, integrated, and cost-effective biological indicator remains imperative for evaluating the relationship between systemic chronic inflammation and cataract pathogenesis.

RAR is an emerging composite biomarker integrating two independent biological indicators reflecting systemic inflammation and nutrition-related status, providing a more comprehensive assessment of homeostatic imbalance. Published literature indicates that RAR is significantly associated with incidence and prognosis of various diseases, including cardiovascular events, diabetic comorbidities, cancer, sepsis, depression and intracerebral hemorrhage.^13^^,^^16^^,^^18^, ^19^, ^20^^,^^41^ In addition, Hao et al. found that elevated RAR was independently associated with increased all-cause and cause-specific mortality in the general population.^42^ Within the field of ophthalmic diseases, RAR was also related to the incidence of age-related macular degeneration and diabetic retinopathy.^17^^,^^43^

To the best of our knowledge, this research is the first to confirm an association between RAR and cataract. We hypothesize that both an elevated RDW and a reduced albumin level may facilitate cataractogenesis. RDW frequently increases under conditions of systemic inflammation and oxidative stress, in which the bone marrow is stimulated to release immature erythrocytes into the peripheral circulation.^44^, ^45^, ^46^ Albumin, the primary circulating protein synthesized by the liver, functions as a "negative acute-phase protein". Albumin production decreases during chronic inflammation and nutritional deficiency, while its uptake and clearance by tissues increase, leading to reduced serum albumin levels.^47^^,^^48^ Pathophysiologically, systemic chronic inflammation elevates inflammatory mediators such as tumor necrosis factor-α (TNF-α). These mediators reach ocular tissues and activate pathways including NF-κB, MAPK, and JAK/STAT3, inducing expression of oxidative stress-related genes while suppressing antioxidant enzymes, such as superoxide dismutase (SOD) and glutathione peroxidase (GP).^49^, ^50^, ^51^, ^52^, ^53^ Reduced albumin levels significantly impair reactive oxygen species (ROS) scavenging capacity of the plasma, weakening systemic antioxidant defenses.^54^ These two homeostatic disruptions synergistically accelerate lens cell damage and protein aggregation, thereby promoting cataract progression.

Our findings demonstrated a positive association between elevated RAR levels and cataract risk. This association remained statistically significant across all regression models, PSM analysis and subgroup analysis, which confirmed the robustness of our conclusions. Subgroup analysis revealed no significant interaction effects between RAR and covariates, while a significant positive correlation between RAR and cataract risk persisted in most subgroups, indicating that the RAR–cataract association was general and not limited to specific populations. Given its cost-effectiveness and ease of measurement, RAR may serve as a routine monitoring marker for cataract. For patients with chronic inflammatory conditions, in addition to managing comorbidities and improving lifestyle, future research should focus on evaluating the potential effects of anti-inflammatory therapies and albumin supplementation for cataract prevention.

This study provides the first investigation into the relationship between RAR and cataract. We utilized a large NHANES dataset spanning all survey cycles containing participant cataract information, ensuring representative sampling. However, limitations warrant acknowledgment. First, the cross-sectional nature of the study precludes further investigation on the causal relationship between RAR and cataract. Second, self-reported surgery history by participants does not accurately reflect the prevalence of cataract, considering the existence of untreated patients and recall bias. Finally, data limitations precluded detailed exploration of associations between RAR and specific cataract types or severity grades. Future research should expand sample sizes through longitudinal multicenter studies to establish causality, while experimental investigations should elucidate the mechanism by which RAR promotes cataract progression, thereby validating its preventive potential.

## 5 Conclusions

This study, utilizing data from 26397 participants in the 1999–2008 NHANES cycles, demonstrates a significant positive association between RAR and cataract development. RAR represents a promising novel biomarker for cataract risk assessment and early prevention, highlighting the critical role of systemic inflammation and nutritional deficiency in cataract pathogenesis. Future research should establish the causal relationship and evaluate potential benefits of targeted interventions addressing these modifiable factors for cataract risk reduction.

## Study approval

The authors confirm that any aspect of the work covered in this manuscript that involved human patients or animals was conducted with the ethical approval of all relevant bodies and the study was performed in accordance with the Declaration of Helsinki, and the protocol was approved by the Ethics Committee of the National Center for Health Statistics (Protocol #98-12 for 1999–2004, Protocol #2005-06 for 2005–2008).

## Author contributions

The authors confirm contribution to the paper as follows: Conception and design of study: RZ, XS; Data collection: RZ; Analysis and interpretation of results: RZ; Drafting the manuscript: RZ, FC, YH, XS; All authors reviewed the results and approved the final version of the manuscript.

## Funding

This work was supported by the National Natural Science Foundation of China (No. 82571186, 82371037), the National Key R&D Program of China (2024YFC2510900/2024YFC2510904).

## Declaration of competing interest

The authors declare that they have no known competing financial interests or personal relationships that could have appeared to influence the work reported in this paper.

## Abbreviations

NHANES: National Health and Nutrition Examination Survey
NCHS: National Center for Health Statistics
RAR: Red blood cell distribution width to albumin ratio
RDW: Red blood cell distribution width
BCP: Bromocresol purple
BMI: Body mass index
CRP: C-reactive protein
IL-6: Interleukin-6
PSM: Propensity score matching
VIF: Variance inflation factor
RCS: Restricted cubic spline
AIC: Akaike information criterion
OR: Odds ratio
CI: Confidence interval
SD: Standard deviation
SII: Ystemic immune-inflammation index
DII: Dietary inflammatory index
TNF-α: Tumor necrosis factor-alpha
SOD: Superoxide dismutase
GP: Glutathione peroxidase
ROS: Reactive oxygen species
